# Comparison of the innovative endoscopic oropharyngeal airway and the conventional mouthpiece in elderly outpatients undergoing esophagogastroduodenoscopy under sedation: a prospective and randomized study

**DOI:** 10.1186/s12876-021-02089-6

**Published:** 2022-01-06

**Authors:** Wei Zhang, Chun Zhu, Xu Chen, Lei Tao, Keqiang He, Hao Wu, Xiaoqing Chai, Sheng Wang, Min Xia

**Affiliations:** 1 Department of Anesthesiology, The First Affiliated Hospital of USTC, Division of Life Sciences and Medicine, University of Science and Technology of China, No. 17, Lujiang Road, Luyang District, Hefei, 230001 Anhui China; 2 Department of Anesthesiology, The People’s Hospital of Sixian County, No. 120, Huayuan Road, Sicheng Town, Suzhou, 234300 Anhui China

**Keywords:** Esophagogastroduodenoscopy, Oropharyngeal airway, sedation

## Abstract

**Background:**

Undesirable outcomes may appear for elderly patients undergoing esophagogastroduodenoscopy (EGD) under sedation, such as hypoxia and hypotension. The aim of our study was to investigate the ability of the innovative endoscopic oropharyngeal airway to reduce the frequency of hypoxia during EGD under sedation in elderly patients.

**Methods:**

In this trial, aged patients undergoing EGD were randomized into airway group and mouthpiece group. The primary outcome was the incidence of the minimum pulse oxygen saturation < 90% and minimum pulse oxygen saturation. In addition, sedation dose, recovery time, emergency management and adverse reactions were recorded.

**Results:**

360 patients completed the study (180 in each groups). The minimum pulse oxygen saturation during EGD was significantly higher in airway group (97.66 ± 2.96%) than in mouthpiece group (95.52 ± 3.84%, *P* < 0.001). The incidence of pulse oxygen saturation of 85–89% of airway group (5.0%, 9/180) was lower than mouthpiece group (10.6%, 19/180, *P* = 0.049). The endoscopy entry time in airway group was 3 (2, 4) seconds and in mouthpiece group was 5 (4, 6) (*P* < 0.001). Propofol total dose and awakening time were significantly lower in the airway group than in the mouthpiece group (*P* = 0.020 and *P* = 0.012, respectively). Furthermore, the incidence rate of hypotension was significantly higher in mouthpiece group (12.2%) than in airway group (5.0%) (*P* = 0.015). By comparison with the mouthpiece group, the satisfaction of endoscopists was higher in airway group (*P* = 0.012).

**Conclusion:**

Elderly patients undergoing EGD, Endoscopy Protector was associated with a significantly lower incidence of hypoxia, shortened endoscopy entry time and more stable hemodynamics.

*Trial registration*: ChiCTR, ChiCTR2000031998, 17/04/2020. http://www.chictr.org.cn/index.aspx

## Background

The frequency of endoscopy under sedation has been growing rapidly for the detection of upper gastrointestinal disease in aging [1]. Although the procedure may be associated with undesirable outcomes, such as upper airway collapse, reflux aspiration, hypoxia, hypotension and arrhythmias, esophagogastroduodenoscopy (EGD) under sedation is still an appropriate option for elderly patients for its advantage of reducing stress and increasing the success rate [2]. Due to the decreased baseline of oxygen tension and frail functions of cardiopulmonary, it is difficult to maintain the stability of respiratory and circulation for elderly patients.

**Fig. 1:**
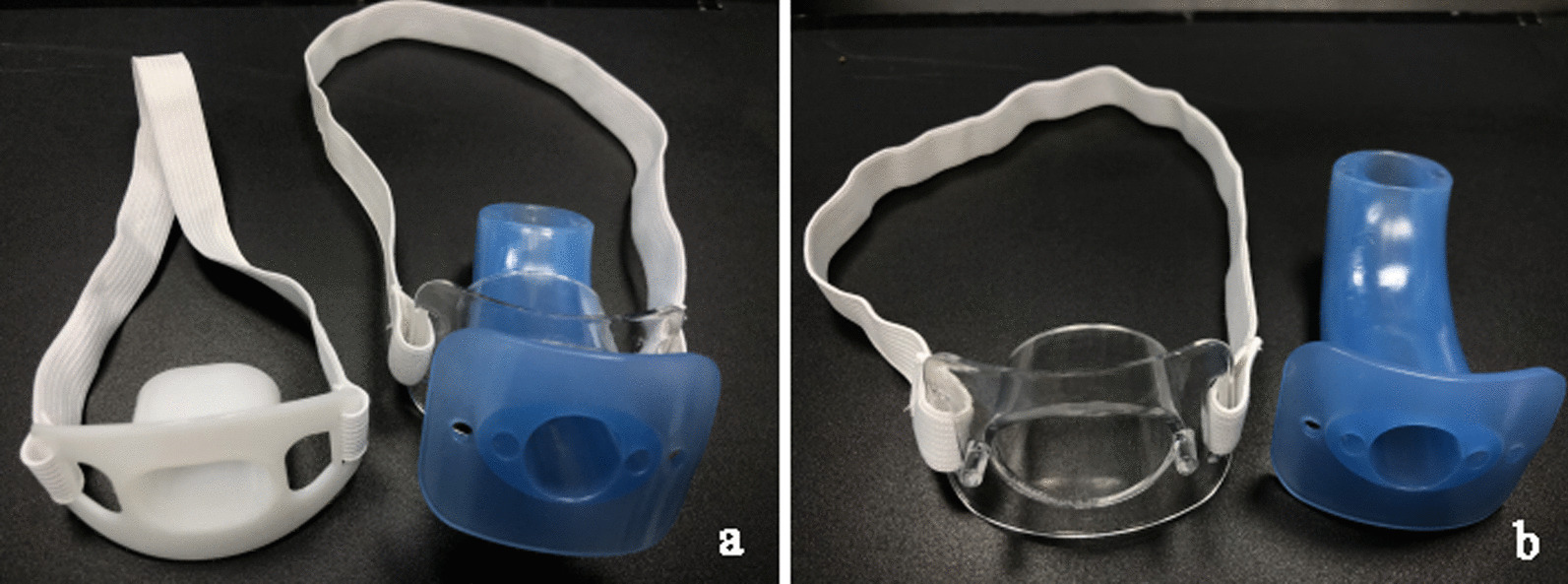
The mouthpiece and the oropharyngeal airway. **a** The conventional mouthpiece (left) and the innovative endoscopic oropharyngeal airway (right). **b** The innovative endoscopic oropharyngeal airway is composed of a transparent mouthpiece (left) and a novel oropharyngeal airway (right)

The main purpose of inserting a conventional mouthpiece during the procedure is to prevent the gastroscope from being bitten by patients (Fig 1). It lacks the function of airway protection. Relaxation of the upper airways musculature and predisposition to upper airway collapse was inevitable due to intravenous anesthesia [3]. The major anatomic site in which the airway obstruction occurs is the pharynx; being the only segment of the upper airway that is not bounded by bony structures [4]. Moreover, the upper airway caliber decreases as people age [5]. Once upper airway collapses or the obstruction occurs, the general approach is to increase oxygen flow immediately, open the airway with the jaw-thrust maneuver, or even withhold the procedure [6, 7]. However, it is inefficient to lift the mandible of patient in the lateral position [8] so that it is useless to implement high-flow oxygen therapy via transoral or transnasal route under such circumstance [9]. Also, reflux or secretions often aggravate hypoxia. As a conclusion, it is an upmost importance to maintain the airway stability and patency during EGD under sedation in elderly patients.

**Fig. 2:**
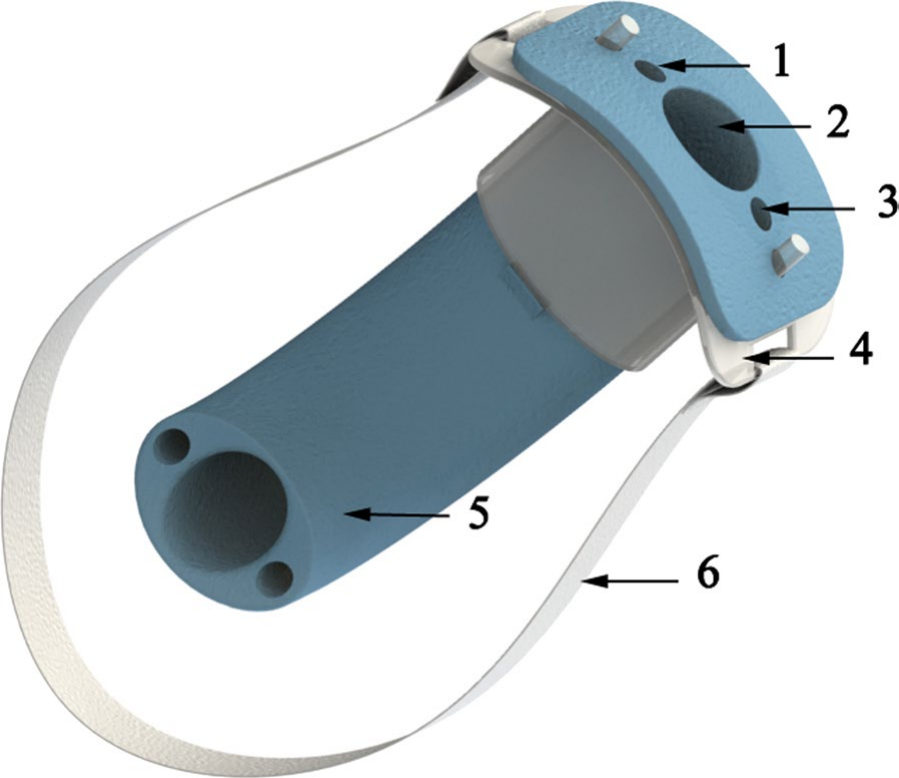
Pictorial depiction of the innovative endoscopic oropharyngeal airway. (1) oxygen supply channel, an oxygen tube is placed to supply oxygen to the pharynx; (2) gastroscope channel, convenient for gastroscope insertion and straight to the pharynx; (3) suction channel, sputum suction tube is placed to suction oropharyngeal secretions; (4) transparent mouthpiece, prevent biting and protect gastroscope; (5) pharyngeal airway, positioned from oral cavity to pharyngeal cavity to avoid airway collapsing; (6) mouthpiece lace

The economic oropharyngeal airway is regularly used to relieve glossocoma in the patients during sedation/general anesthesia [10]. The mechanism of this effect is explained by the improvements in the velopharyngeal and oropharyngeal airways, which are induced by mandibular advancement [11]. However, it is not suitable for upper endoscopy for the reason that it is too slim and unsteady to hold the gastroscope. The modified endoscopic oropharyngeal airway is commercialized as Endoscopy Protector, which is composed of a transparent mouthpiece and a novel oropharyngeal airway (Figs. 1, 2). It effectively prevents a patient from involuntarily biting endoscope and avoids a patient’s airway from collapsing. This present study sought to determine the clinical efficiency of the innovative airway in EGD under sedation. We hypothesized that the innovative endoscopic oropharyngeal airway could reduce the chance of hypoxia in elderly patients undergoing endoscopy.

## Methods

### Patients

The study enrolled patients scheduled for EGD under sedation at the outpatient department of the first affiliated hospital of USTC between April 2020 and December 2020. The indications of upper endoscopies were gastroesophageal reflux, abdominal pain, gastrointestinal bleeding and anemia. All subjects conformed to the following inclusion criteria: male or female patients; age between 65 and 80 years; American Society of Anesthesiology (ASA) score I–II physical status; and ability to fill out a survey form and give informed consent. Exclusion criteria were: psychiatric and neurological history; severe cardiovascular or pulmonary diseases; body mass index (BMI) > 30 kg/m^2^; sleep apnea; impaired kidney or liver function; long-time opioid or antipsychotic medication history; acute upper gastrointestinal bleeding; allergic to propofol or the emulsifier content; and an expected upper endoscopy duration more than 30 min. This protocol was approved by the Medical Ethics Committee of the First Affiliated Hospital of University of Science and Technology of China (USTC). All patients gave their written informed consent. The study was conducted according to the Declaration of Helsinki.

All the investigators and the anaesthetists participated in standardized training for the operation of the innovative endoscopic oropharyngeal airway, including the mechanism of action for its use in EGD, and management of the adverse events. Based on a random number table generated by a computer, eligible patients were randomized in a 1:1 ratio to two groups with block sizes of 2: mouthpiece group or airway group. The patients were unaware of group assignment.The randomization sequence was kept in sealed opaque, identical envelopes. The envelopes were opened just before preparation of the mouthpiece or airway. Dosing of anesthetic medications was at the discretion of the anaesthetists. Outcome assessment was performed by the investigator. One of the investigators placed the device and managed airway.

### Sedation methods

All patients were fasted for 8 h before the upper endoscopy. 10 ml of dyclonine glue was taken orally 30 min before EGD. The venous channel was established in the upper limb. The subjects were placed in the left lateral decubitus position. Electrocardiography, pulse oxygen saturation, heart rate and blood pressure (cuff placed on the left upper arm) were continuously monitored during the procedure. A conventional mouthpiece was used in mouthpiece group for endoscope, while placed an innovative endoscopic oropharyngeal airway commercialized as Endoscopy Protector in airway group (45–70 kg, medium size; 60–100 kg, large size) (Dami Medical Technology Co.,Ltd, Hefei, China.). The nasal catheter was placed in the two groups before the venous anesthesia and both groups were given oxygen at the same rate of 5 L/min for 1 min.

**Fig. 3:**
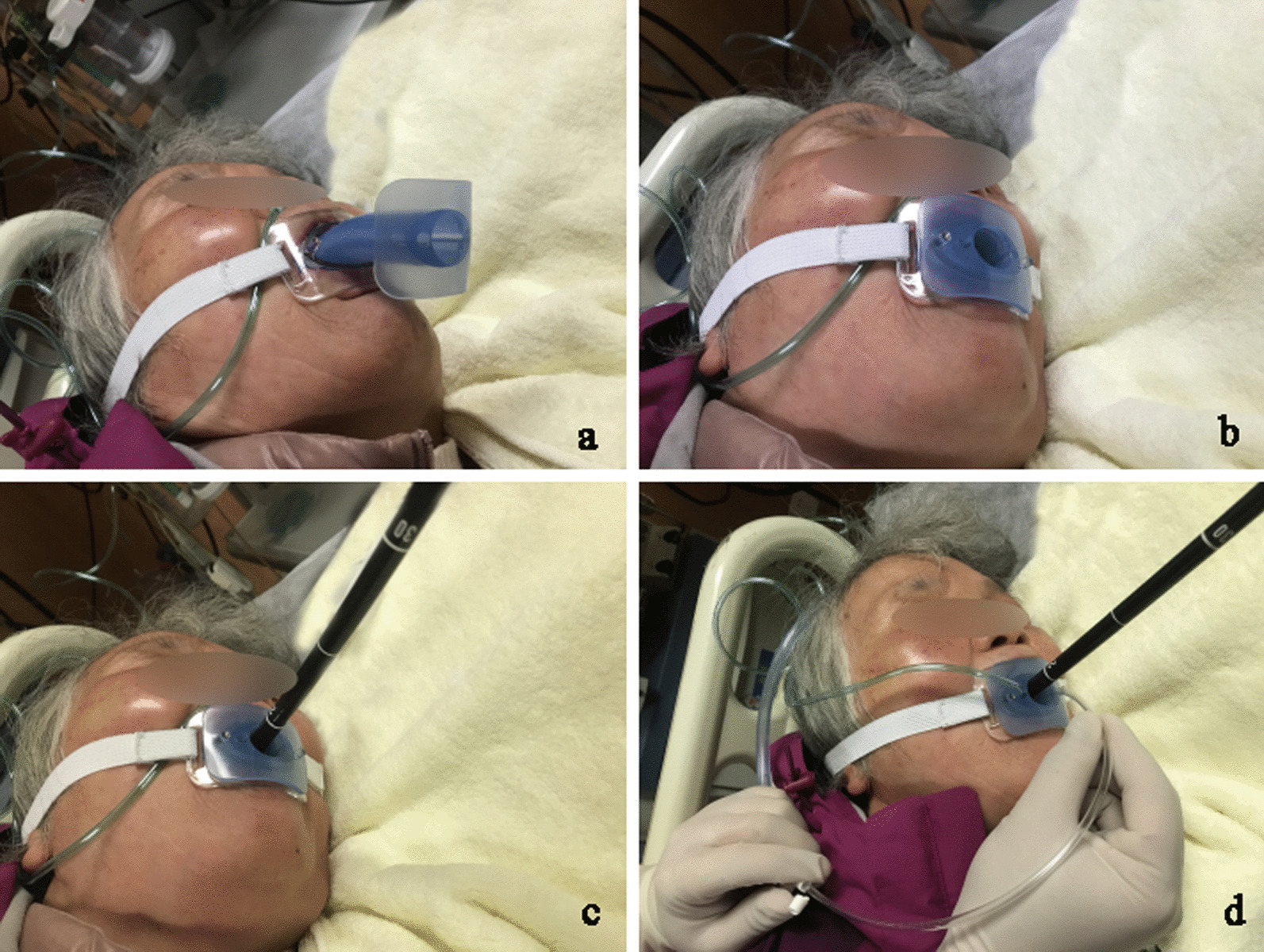
Application of the innovative endoscopic oropharyngeal airway in an aged outpatient undergoing esophagogastroduodenoscopy under sedation. **a** ready to insert the novel oropharyngeal airway; **b** the novel oropharyngeal airway was inserted into the transparent mouthpiece; **c** the gastroscope was inserted through the main channal of the novel oropharyngeal airway; **d** suction secretions and reflux through the special suction channel during the procedure

Patients in two groups were given intravenous 1.0% propofol (batch number: 1912296; Fresenius Kabi, Graz, Austria) at an induction dose of 1.5 mg/kg over a 1 min period followed by a maintenance dose at 2–5 mg/kg/h [2]. EGD was performed after the patient’s consciousness disappeared and the eyelash reflex disappeared. After the nasal oxygen tube was placed from nostril to the mouthpiece, the subjects in mouthpiece group received the gastroscope. In the airway group, after the novel oropharyngeal airway being soaked in normal saline for 10 s for the super-slip material on the surface being activated, it was inserted into the transparent mouthpiece (Fig. 3). And the nasal oxygen tube was placed from nostril to the airway channel of the novel oropharyngeal airway and the upper endoscopy began (Fig. 3). All the procedures were performed by the same endoscopist. Spontaneous respiration was maintained during the procedure.

### Monitoring and management of adverse events

All patients undergoing EGD under sedation were evaluated according to the Ramsay Sedation Scale (RSS) [12], which was maintained at > 4 during upper endoscopy. If a patient showed be in discomfort or exhibited restlessness, an additional 10 mg of propofol was given as a bolus injection and the maintenance infusion rate was increased by 1 mg/kg/h. If an adverse event was appeared, the dose of maintenance sedation was decreased. Under the supply of 100% O_2_ 5 L/min via nasal cannula, when the patient’s blood oxygen saturation was less than 90%, the mandible was lift up to maintain upper airway patency and the oxygen flow was enhanced. When the patient’s blood oxygen saturation was less than < 85%, suspend endoscopy and quickly pulled out gastroscope to hold up the oxygen mask to assist breathing. Tracheal intubation for mechanical ventilation was performed if necessary. When the sound of airflow through secretions were found or choke occurred during the procedure, sputum suction were recommended for immediate implementation. The effective suction secretion and reflux was defined as that airway was patency and pulse oxygen saturation could be promoted after suction. Subclinical respiratory depression: if pulse oxygen saturation of 90–94% [13]. Hypoxemia: desaturation events were defined as mild (if pulse oxygen saturation of 85–89%) and moderate (if pulse oxygen saturation < 85%) [14, 15]. Bradycardia: if the heart rate is less than 50 bpm during EGD, a 0.5 mg dose of atropine were given intravenously. Hypotension: if the systolic blood pressure drops more than 20% of the baseline or less than 90 mmHg, 20 μg dose of phenylephrine was given.

### Assessments

Heart rate (HR), mean arterial pressure (MAP) and pulse oxygen saturation of patients were recorded before anesthesia, during EGD and after EGD. The recorded pulse oxygen saturation during EGD was minimum value in the period. Endoscopy entry time, first successful entry rate, duration of endoscopy, the dose of sedation and recovery time were recorded. The incidence of emergency management and adverse reactions were recorded. Airway interventions during procedure (including lifting the mandible, hyperbaric oxygen supply, pause Endoscopy, assisted ventilation and gastroscope withdraw) were recorded.

The novel oropharyngeal airway was taken away after the gastroscope was pulled out from esophagus in airway group. The patient was transferred to the PACU (post-anesthesia care unit) after the procedure was completed. The transparent mouthpiece and conventional mouthpiece were also taken out after the patients became conscious. The subjects were followed up 1 h later, receiving an Aldrete score after anesthesia with a score of 9 or higher [16], and may be discharged with the company of relatives and friends. The satisfaction of the physician and patient were assessed using a 10-point scale as follows: poor, 1–4; fair, 5–7; good, 8–10.

The primary outcome was the incidence of the minimum pulse oxygen saturation < 90% and minimum pulse oxygen saturation. The secondary outcomes were endoscopy entry time, first successful entry rate, duration of upper endoscopy, recovery time, sedation dose (propofol) and the incidences of adverse reactions. The adverse reactions were tongue retraction, cough, body movements, hiccups, reflux and aspiration. The recovery time includes the awakening time and the time from awakening to leave. The awakening time is defined as the time from the end of the procedure to the acquisition of an Aldrete score of 9 or higher.

### Sample size calculation

A previous study showed that the minimum pulse oxygen saturation < 90% in aged patients using nasal catheter and a conventional mouthpiece undergoing upper endoscopy under sedation was 15.9% and a reduction to 6.2% respectively using endoscopic mask [7]. Based on our pilot study, we assumed the 14.0% incidence of the minimum pulse oxygen saturation < 90% in aged patients using a conventional mouthpiece and a clinically important reduction to 5.0% happened with using the innovative endoscopic oropharyngeal airway. The required sample size was calculated to be 166 patients per group (α = 0.05 and β = 0.2). To account for potential dropouts, 366 patients were recruited. Analysis was conducted on an intention-to-treat basis.

### Statistical analysis

Data were presented as means ± standard deviation for normally distributed data and median and range for skewed data. Absolute numbers were presented as percentages of participants. Depending on the distribution of the data, continuous variables were compared using a 2-tailed Student t test or Mann–Whitney U test. Categorical variables were compared using the Pearson chi-square test or Fisher exact test. A *P* < 0.05 was considered statistically significant. For statistical analysis, we used SPSS version 24.0 (SPSS Inc., Chicago, IL).

## Results

### Patient background

**Fig. 4:**
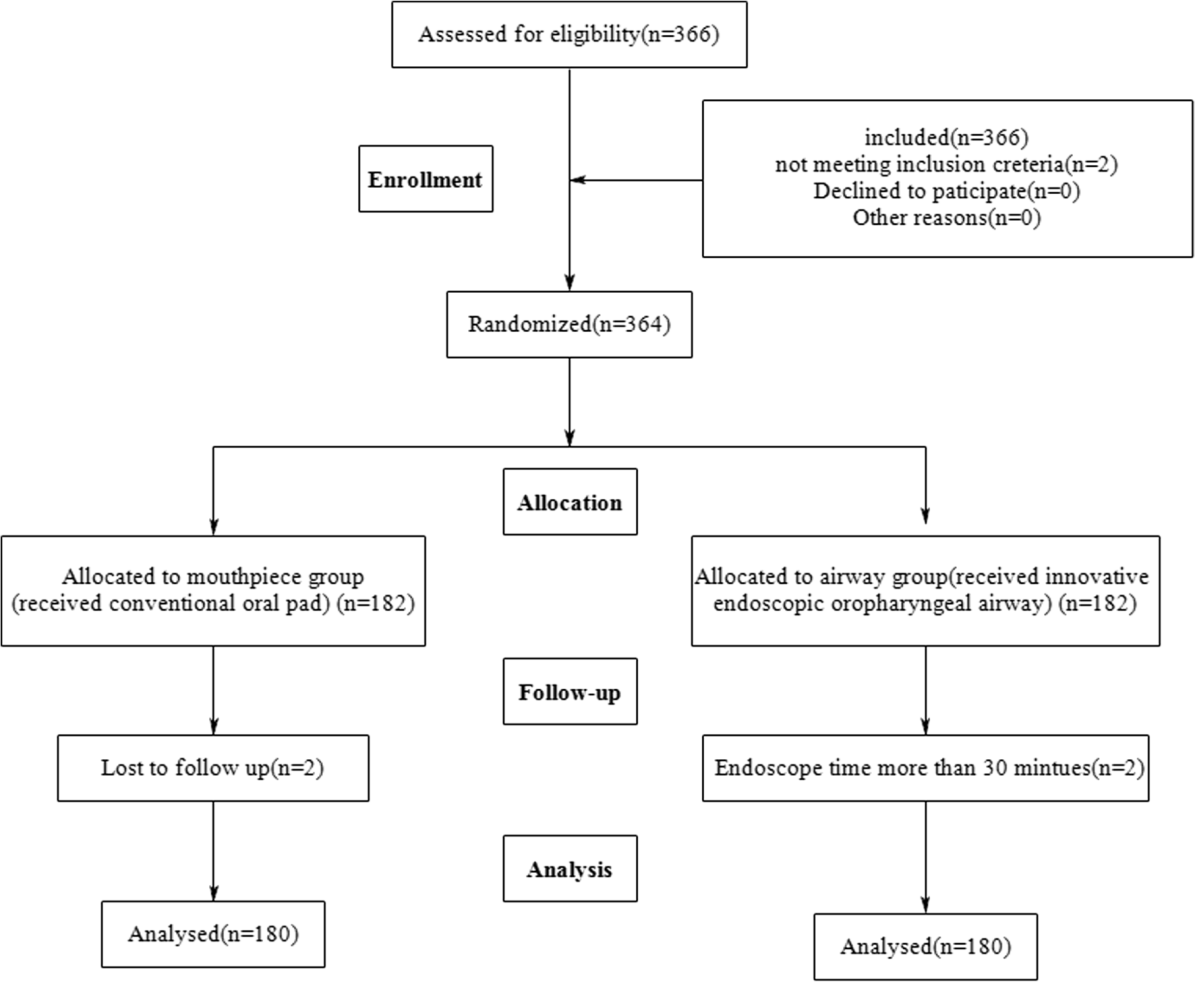
The flow chart of participant selection for painless gastroscopy

Initially, 366 patients were assessed. Six patients were excluded from the study. Two did not meet the inclusion criteria. Two were lost to follow for leaving the PACU without permission. And two endoscopy time was more than 30 min due to removing gastric polyps. Therefore, 360 patients completed the study, with 180 in each groups (Fig. 4). The baseline characteristics were not significantly different in terms of age, sex, body mass index, alcohol and smoking history, American Society of Anesthesiologists (ASA) risk score and underlying diseases in two groups (Table 1).

**Table 1:** Demographic and clinical characteristics of aged patients undergoing EGD under sedation

	Mouthpiece group (n = 180)	Airway group (n = 180)	*P-*value
Age (year)	69.87 ± 3.94	69.19 ± 3.73	0.92
Sex (male/female)	97/83	92/88	0.598
Weight (kg)	62.69 ± 9.41	61.87 ± 10.22	0.427
Height (cm)	164.81 ± 7.36	164.42 ± 7.67	0.629
BMI (kg/m^2^)	23.05 ± 2.92	22.81 ± 2.84	0.416
Smoking history (%)	16 (8.9)	19 (10.6)	0.594
Alcohol history (%)	11 (6.1)	14 (7.8)	0.534
Allergy (%)	0 (0.0)	0 (0.0)	NA
Chronic obstructive pulmonary disease (%)	0 (0.0)	1 (0.6)	0.317
Diabetes (%)	3 (1.7)	4 (2.2)	0.703
Hypertension (%)	28 (15.6)	27 (15.0)	0.884
Arterial coronary disease (%)	2 (1.1)	4 (2.2)	0.410
Cerebrovascular disease (%)	3 (1.7)	3 (1.7)	NA
Modified Mallampati Score (I/II/III)	78/98/4	85/93/2	0.577
ASA risk score (I/II)	57/123	66/114	0.317

Values are means standard deviation or n (%)

ASA, American Society of Anesthesiologists; BMI, body mass index. NA-not applicable due to low event rate

### Respiratory depression

Primary outcome is shown in Table 2. The incidence of pulse oxygen saturation of 85–89% of airway group (4.4%) was lower than in mouthpiece group (12.2%, *P* = 0.008; Table 2).

**Table 2:** Pulse oxygen saturation and emergent management

	Mouthpiece group (n = 180)	Airway group (n = 180)	*P*-value
Pulse oxygen saturation			
Subclinical respiratory depression,pulse oxygen saturation of 90–94% (%)	19 (10.6)	9 (5.0)	0.049
Mild hyoxemia, pulse oxygen saturation of 85–89% (%)	22 (12.2)	8 (4.4)	0.008
Moderate hyoxemia, pulse oxygen saturation < 85% (%)	2 (1.1)	0 (0.0)	0.156
Emergency management			
Lifting the mandible (%)	25 (13.9)	11 (6.1)	0.014
Enhancing oxygen flow (%)	43 (23.9)	17 (9.4)	0.000
Withholding endoscopy (%)	17 (9.4)	6 (3.3)	0.018
Effective suction secretions and reflux (%)	5 (2.8)	15 (8.3)	0.021
Removing the endoscopy tube and mask ventilation (%)	2 (1.1)	0 (0.0)	0.156

Values are presented as n (%)

### Pulse oxygen saturation, MAP or HR

**Fig. 5:**
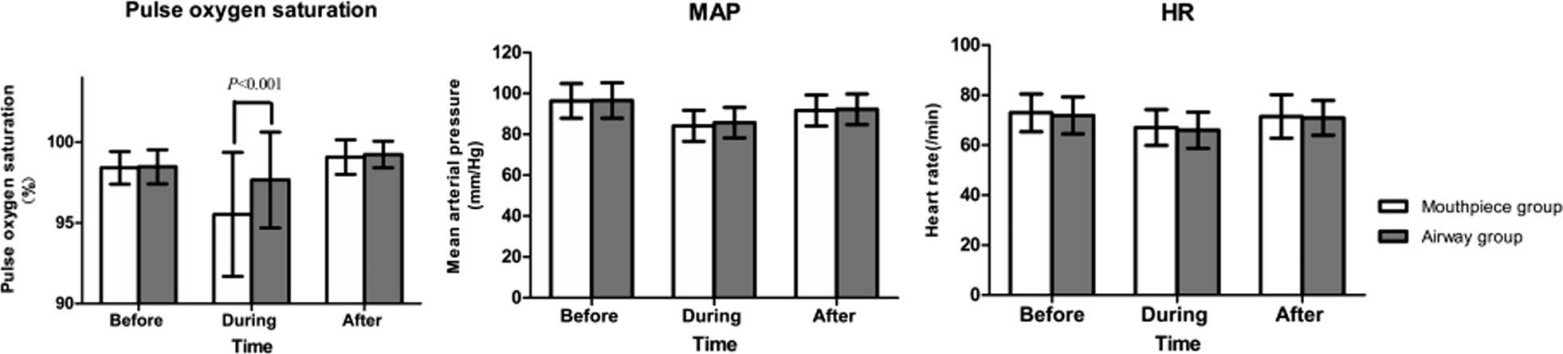
Pulse oxygen saturation (SpO_2_), mean arterial pressure (MAP) and heart rate (HR) of aged patients. The SpO_2_ during EGD under sedation was higher in the airway group than in the mouthpiece group among aged patients (*P < 0.001). MAP and HR between the airway group and the mouthpiece group showed no difference (*P* > 0.05)

The pulse oxygen saturation during endoscopy under sedation was significantly higher in airway group (97.66% ± 2.96%) than in mouthpiece group (95.52% ± 3.84%, *P* < 0.001; Fig. 5).

### Procedure time and sedation dose

Endoscopy entry time in airway group was 3 (2, 4) seconds and in mouthpiece group was 5 (4, 6) seconds (*P* < 0.001). Propofol induction dose were significantly lower in mouthpiece group than in airway group (*P* = 0.042). In addition, propofol additional dose, propofol total dose and awakening time were significantly lower in airway group than in mouthpiece group (*P* < 0.001, *P* = 0.020 and *P* = 0.012, respectively; Table 3)*.*

**Table 3:** Endoscopy performance, recovery time and sedation dose in two groups

	Mouthpiece group (n = 180)	Airway group (n = 180)	*P*-value
Endoscopy performance			
Endoscopy entry time (second)	5 (4,6)	3 (2,4)	0.000
First successful entry rate (%)	162 (88.9)	173 (96.1)	0.023
Duration of endoscopy (second)	380.23 ± 55.10	371.18 ± 59.51	0.135
Recovery time			
Awakening time (second)	182.89 ± 80.48	163.11 ± 67.30	0.012
Time from awakening to leave (second)	1057.50 ± 193.67	1040.33 ± 176.76	0.380
Sedation dose			
Propofol indutcion dose (mg)	98.28 ± 13.86	101.11 ± 12.46	0.042
Propofol additional dose (mg)	20 (10.40)	10 (10.30)	0.000
Propofol total dose (mg)	123.78 ± 22.10	118.61 ± 19.91	0.020

Values are means ± standard deviation, median (interquartile range) and n (%)

### Sedation-related complications

Similarly, the incidence rate of hypotension and body movements was significantly higher in mouthpiece group (12.2% and 11.7%) than in airway group (5.0% and 5.6%) (*P* = 0.015 and 0.039; Table 4). In contrast, incidence of palate injury, nausea/vomiting, hiccup or arrhythmia and satisfaction of the patient between airway group and mouthpiece group showed no difference (all *P* > 0.05; Table 4). Compared with mouthpiece group, effective suction secretions and reflux, and the satisfaction of the endoscopist was higher in airway group (*P* = 0.021 and 0.012; Table 4).

**Table 4:** Adverse reaction and the satisfaction in two groups

	Mouthpiece group (n = 180)	Airway group (n = 180)	*P*-value
Adverse reaction			
Palate injury (%)	0 (0.0)	0 (0.0)	NA
Hypotention (%)	22 (12.2)	9 (5.0)	0.015
Nausea/vomiting (%)	4 (2.2)	5 (2.8)	0.736
Cough (%)	15 (8.3)	8 (4.4)	0.131
Body movements (%)	21 (11.7)	10 (5.6)	0.039
Body movements, general (%)	18 (10.0)	9 (5.0)	0.072
Body movements, serious (%)	3 (1.7)	1 (0.6)	0.315
Hiccup (%)	3 (1.7)	1 (0.6)	0.315
Arrhythmia (%)	7 (3.9)	3 (1.7)	0.200
The satisfaction			
The satisfaction of endoscopist (good/fair/poor)	128/42/10	148/30/2	0.012
The satisfaction of patient (good/fair/poor)	108/60/12	121/55/4	0.084

Values are means ± standard deviation or n (%)

NA-not applicable due to low event rate; Poor, 1–4; fair, 5–7; good, 8–10

## Discussion

Our study showed that the application of the innovative endoscopic oropharyngeal airway in aged patients could increase the minimum pulse oxygen saturation during upper endoscopy and provide more stable hemodynamics without increasing examination time. In addition, shorter endoscopy entry time and faster awakening time appeared promising.

For maintaining airway stability and patency in EGD, the main problem we had to deal with was to take precautions against tongue retraction and expand reduced pharyngeal space with airway collapsibility [1]. Some devices placed above the glottis have been developed to reduce the incidence of hypoxia, such as the novel endoscopic mask [17, 18], reflux-preventing face mask [19], nasopharyngeal airway [20], nasopharyngeal catheter [21], dual channel laryngeal mask airway [22], high-flow nasal cannula [23], supraglottic jet oxygenation and ventilation [13], et al. Although the modified endoscopic airway devices yet have been useful in reducing the risk of hypoxia, they may have a few disadvantages. The new endoscopic mask is composed of a mask and an oropharyngeal airway, which are closely connected [18]. It is difficult for the anesthetist to adjust the position of oropharyngeal airway during the procedure. The position of the novel mask will be inevitably moved during EGD, which will affect the ventilation effect [17, 18]. There has not been any prospective study to prove the advantage of reflux-preventing face mask in the EGD under sedation and further investigations are required [19]. The mucosa of the nasal cavity could be damaged by the nasopharyngeal airway and nasopharyngeal catheter, and bleeding also often occurred [20, 21]. In addition, due to the space of the nasal cavity, which is less than the oral cavity, hypoxia is indeed improved but with limited effect. Laryngeal mask airway has been proved to be effective on the improvement of airway and ventilation management in upper gastrointestinal endoscopy [22]. On the other hand, the size of the new dual channel laryngeal mask airway is too big that more sedation is needed to achieve the depth of anesthesia for insertion. The previous study showed that propofol of 400 (280–540) mg was consumed in EGD [22], which is more than usual. The excessive sedation will weaken the efficiency of the procedure. In addition, sore throat is a common postoperative complication. The method of increasing the inhaled oxygen flow has been proved to have a favorable risk-to-benefit ratio, such as HFNC (high flow nasal cannula) [23] and supraglottic jet oxygenation and ventilation [13]. However, with the assistance of the supportive oxygen therapy, we still need emergency management to keep the airway patency, such as lifting the mandible. The above two devices often cause postoprocedure xerostomia and flatulence; it creates discomfort for patients post-sedation. The ideal airway device for EGD under sedation should be portable, economic and with minimum complication; it should be easy to maintain the stability and patency of upper respiratory tract as well.

Compared with the conventional oropharyngeal airway, several structural modifications of novel airway have been made (Figs. 2, 3). It is similar as a utility of modified oropharyngeal airway for performing tracheal intubation using a fiberoptic bronchoscope by Lee et al. [24]. The primary channel of the innovative airway is to allow the gastroscope being put through and prevent the upper airway from collapsing. Unlike the modified oropharyngeal airway by Lee et al. [24] which is half-sealed, the novel airway is designed to be a sealed tubular with an inner diameter of 18 mm due to the diameter of gastroscope is twice the size of fiberoptic bronchoscope so that it allows the gastroscope to rotate with ease. The surface of the novel airway is also covered by the special polyester to increase lubrication for gastroscope insertion. There are two small channels with 5 mm inner diameter extending to the distal end on both sides of the main channel—one for quick sputum suction and the other for effective oxygen inhalation. It is well known that aged individuals exhibit an impaired pharyngeal function, incoordination of swallowing and breathing [25]. Elderly patients generally have a reduction in pharyngeal sensitivity leading to higher risk of aspiration. Our study indicated that the number of effective suction secretions in the airway group was higher than the mouthpiece group’s.

Kim et al. [10] observed size-9 and size-8 oropharyngeal airways (Guedel) were the appropriate sizes for clinical use in Korean men and women of average height, respectively. The length of the airway was measured from the flange to the distal end by passing the fiberscope through the channel of the airway (size 9 = 10 cm, size 8 = 9 cm). Under the consideration of the physical characteristics of Asians, we have designed the novel airway by measuring the oropharyngeal dimensions—soft palate, the dorsum of the tongue and pharyngeal wall [26]. The distal end of the innovative airway is made to be set about 2 to 3 cm away from esophageal entrance. Effect of vagal stimulation, pharyngeal collapsing, tongue retraction and endoscopy operation are the primary issues among all factors. The large size (8.5 cm, green) and medium size (8.0 cm, blue) are selected, which are 0.5–1.0 cm shorter than the conventional airway. The actual length from the flange to the distal end through the channel (large size 9 = 10.5 cm, medium size = 9.5 cm) is 0.5 cm longer than the conventional airway. The radian of innovative airway is slightly higher than the conventional airway, which is convenient for endoscopist to find the esophageal entrance and rotate the gastroscope freely. It is identified in our study that there were lower endoscopy entry time and higher first successful entry rate in airway group than those of mouthpiece group. It was necessary to note that emergency management was less likely to be involved and the risk of mild hypoxia went down significantly (pulse oxygen saturation of 85–89%) (4.4%) under the innovative airway treatment.

A previous trial has shown that endoscopy under sedation was safer, more comfortable and effective for chronic hypertension patients [27]. Seniors with chronic hypertension have a higher risk of hypotension during sedation which will increase the incidence of cerebrovascular disease. Although propofol is world-widely used for standard endoscopy procedures, oxygen desaturation and hypotension remain drawbacks [28, 29], so that it is necessary to avoid overdosing and conduct management with caution during procedure [30]. The novel airway may make a special contribution to hemodynamic stability. The nerve in the root of the tongue was so abundant that more sedations were needed to reduce the entrance and irritation of gastroscope [31]. In the airway group, it needed more induction dose of propofol for device insertion, the maintaining sedation had been lowered because the application of innovative airway keeps the contact with gastroscope and oropharynx wall along with any irritation at a minimum [32]. In our study, the total dosage of sedation in the airway group was lower [33]. The results in the current study also demonstrated that the incidence rate of hypotension and body movements was significantly lower in airway group (5.0% and 5.6%) than in mouthpiece group (12.2% and 11.7%). Our observations were in line with the previous finding—the less anesthetics, the lower the incidence of hypotension. As a result, it leaded to a shorter awakening time [34]. In the present study, endoscopists' satisfaction in airway group was higher than the endoscopists' in mouthpiece group for the non-problematical procedure.

There are limitations to our study. For one, hemodynamic changes may be affected by a few factors, for instance, vagal stimulation is likely to occur when the distal end of the novel oropharyngeal airway reaches up to the posterior pharyngeal wall. Although the incidence of hypotension was significantly higher in mouthpiece group, we could fail to notice that hypotension possible immediately appeared after the induction, especially in airway group. Also, it is a bias about the method of sedation maintaining owning to the differences in tolerance or threshold among anesthesiologists. The definition methods of the effective suction secretion and reflux in the present research may be flawed. However, there is no objective relevant parameter. The novel oropharyngeal airway was inserted after patients lost consciousness, and all were removed before the patients awoke. The patients was blinded to group assignment. It would be difficult for patients to deduce their group allocation correctly. However, due to the close distance between the anaesthetist and the investigator, they could easily guess which group the patients were allocated. The higher propofol induced dosage in the airway group also suggested the “blinding” anaesthetist. Therefore, we believed that our study was a single-blinded protocol [13]. The anaesthetist was not blinded, which might have resulted in bias and could have affected the power of this study. However, the objective parameter of hypoxia was the main outcome in the present study, which might correct the single-blindness. In addition, no capnographic monitoring was used because it can’t reduce severe hypoxia during sedation in gastroscopy [35]. We have confidence that capnographic monitoring catheter in the exterior wall of the novel oropharyngeal airway will be made, which may improve the earlier detection of suppressed or apnoeic breathing [36].

## Conclusion

The use of Endoscopy Protector yielded the advantages of preventing desaturation and shortening the endoscopy entry time, along with a stabilizing hemodynamics when elderly patients were undergoing EGD. We believe the innovative airway is closer to the ideal proposed device for EGD.

## Data Availability

The datasets used and/or analyzed during the current study are available from the corresponding author on reasonable request.

## Abbreviations

EGD: Esophagogastroduodenoscopy
ASA: American Society of Anesthesiology
USTC: University of Science and Technology of China
RSS: Ramsay Sedation Scale
HR: Heart rate
MAP: Mean arterial pressure
SpO_2_: Pulse oxygen saturation
